# Adenine Nucleotide Metabolites in Uremic Erythrocytes as Metabolic Markers of Chronic Kidney Disease in Children

**DOI:** 10.3390/jcm10215208

**Published:** 2021-11-08

**Authors:** Joanna Piechowicz, Andrzej Gamian, Danuta Zwolińska, Dorota Polak-Jonkisz

**Affiliations:** 1 Department of Medical Biochemistry, Wroclaw Medical University, 50-556 Wroclaw, Poland; joanna.piechowicz@onet.pl; 2 Hirszfeld Institute of Immunology and Experimental Therapy, Polish Academy of Sciences, 50-556 Wroclaw, Poland; andrzej.gamian@hirszfeld.pl; 3 Department of Pediatric Nephrology, Wroclaw Medical University, 50-556 Wroclaw, Poland; danuta.zwolinska@umed.wroc.pl

**Keywords:** adenine nucleotide metabolites, chronic renal failure, children

## Abstract

Chronic kidney disease (CKD) is associated with multifaceted pathophysiological lesions including metabolic pathways in red blood cells (RBC). The aim of the study was to determine the concentration of adenine nucleotide metabolites, i.e., nicotinamide adenine dinucleotide (NAD)-oxidized form, nicotinamide adenine dinucleotide hydrate (NADH)-reduced form, nicotinic acid mononucleotide (NAMN), β-nicotinamide mononucleotide (NMN), nicotinic acid adenine dinucleotide (NAAD), nicotinic acid (NA) and nicotinamide (NAM) in RBC and to determine a relationship between NAD metabolites and CKD progression. Forty-eight CKD children and 33 age-matched controls were examined. Patients were divided into groups depending on the CKD stages (Group II-stage II, Group III- stage III, Group IV- stage IV and Group RRT children on dialysis). To determine the above-mentioned metabolites concentrations in RBC liquid chromatography-mass spectrometry was used. Results: the only difference between the groups was shown concerning NAD in RBC, although the values did not differ significantly from controls. The lowest NAD values were found in Group II (188.6 ± 124.49 nmol/mL, the highest in group IV (324.94 ± 63.06 nmol/mL. Between Groups II and IV, as well as III and IV, the differences were statistically significant (*p* < 0.032, *p* < 0.046 respectively). Conclusions. CKD children do not have evident abnormalities of RBC metabolism with respect to adenine nucleotide metabolites. The significant differences in erythrocyte NAD concentrations between CKD stages may suggest the activation of adaptive defense mechanisms aimed at erythrocyte metabolic stabilization. It seems that the implementation of RRT has a positive impact on RBC NAD metabolism, but further research performed on a larger population is needed to confirm it.

## 1. Introduction

Chronic kidney disease (CKD), along with cardiovascular diseases, obesity or diabetes, belongs to diseases of affluence. CKD is a challenge for the medical world in the 21st century not only due to the increase in the number of cases, overburdens incurred by prevention measures concerning the development of the disease and its accompanying complications, but also due to the search for methods of its early diagnosis [1].

The origin of CKD is associated with multifaceted pathophysiological lesions including i.a. arginine-creatine metabolic pathways, arginine methylation, urea cycle or glycolytic pathways. Such metabolic pathway disorders are co-responsible for changes in the concentration of various metabolites determined e.g., in patients’ blood, also important for the diagnosis of disease processes [2,3,4,5]. Therefore, according to Cisek et al., Markers identified by ‘omics’ research technologies (metabolomics, proteomics, transcriptomics) can improve not only the prediction of the development of various diseases (including CKD) but will even allow the development of personalized therapy [6].

Although CKD is a global health problem, little information is available concerning metabolomics in the pediatric patient population. For pediatrics and neonatology, according to Mussap et al., metabolomics offers new perspectives in the treatment of sick children, allows for early diagnosis of metabolic profiles associated with the development of the disease, as well as provides personalised therapy for this population [7]. This is of great importance for children because all chronic diseases lead to the inhibition of the body’s growth along with irreversible processes of hormonal imbalance, bone or cardiovascular lesions and the development of hypertension. Currently, the mechanism of CKD development at the molecular level is still not fully understood.

Thus, NAD plays a key role in biological processes related to the response to cellular or genotoxic stress and participates in the metabolism of carbohydrates and fats through SIRT1 activity [8,9,10,11,12]. In the human body, NAD can be synthesized de novo or by a ‘salvage pathway’ (‘recovery process’). The energy metabolism of the red blood cell is mainly based on glycolysis.

Reduced concentrations of NAM and NMN can be explained by an impaired “*recovery process*” in erythrocytes or weakened NAM incorporation due to cell membrane deformation when deficiency of ATP leads to dehydration of cells and their spiky shape [4,13,14,15].

The debate on pathophysiology and biochemistry of processes accompanying kidney damage during CKD development remains open. The identification and validation of the analytes of the ongoing processes will contribute significantly to the understanding of CKD pathomechanisms, the development of accurate prevention principles and the implementation of innovative, effective therapies.

The aim of the study was to determine the concentration of adenine nucleotide metabolites, i.e., nicotinamide adenine dinucleotide (NAD)-oxidized form, nicotinamide adenine dinucleotide hydrate (NADH)-reduced form, nicotinic acid mononucleotide (NAMN), β-nicotinamide mononucleotide (NMN), nicotinic acid adenine dinucleotide (NAAD), nicotinic acid (NA) and nicotinamide (NAM) in erythrocytes of children with chronic kidney disease and to establish a relationship between the concentrations of these NAD metabolites and the development of CKD.

## 2. Materials

### 2.1. The Study Group

The study included 48 patients with chronic kidney disease (16 girls and 32 boys) aged 3–18 years (mean age 11.00 ± 4.72 years) treated in the Department of Pediatric Nephrology and Dialysis Station of the University Hospital.

All the respondents met the inclusion criteria which were:

- Age of 3–18 years,
- Diagnosed CKD of varying degrees of progression
- And written consent to participate in the study.

Patients meeting the exclusion criteria, i.e., failure to meet the inclusion criteria, such as recognition of another acute/chronic inflammatory disease, lack of cooperation and/or abnormalities that may affect the course of the research procedure, were not eligible for the study.

Taking GFR (glomerular filtration rate) values into account (estimated on the basis of Schwartz formula: eGFR (mL/min per 1.73 m^2^) = 0.413 * [height (cm)/serum creatinine (mg/dL)]), groups corresponding to a given stage of disease progression have been distinguished among CKD patients [16,17].

The size of groups in each stage of CKD is as follows:

Group II—15 patients with stage II CKD; including 11 boys, 4 girls;
Group III—16 patients with stage III CKD; including 10 boys, 6 girls;
Group IV—8 patients with stage IV CKD; including 4 boys, 4 girls;
Group of children undergoing renal replacement therapy (RRT)—9 patients undergoing.RRT (hemodialysis, peritoneal dialysis); including 7 boys, 2 girls.

The cause of chronic kidney disease (CKD) in the studied population of patients was glomerulonephritis (18), pyelonephritis (20), and congenital defects of the urinary tracts (10). The hemodialysis [3–4 sessions per week (3–3.5 h)] was applied in 4 children using polysulfone membranes, NaHCO_3_-buffered dialysate, Ca^+2^ content—1.25 or 1.5 mmol/L. Peritoneal dialysis applied in 5 children includes NIPD—nocturnal intermittent peritoneal dialysis and CCPD—continuous cycling peritoneal dialysis with Baxter’s Home Choice using 1.36% or 2.27% Physioneal. The duration of renal replacement therapy (RRT) is 2.02 ± 0.51 years.

In pediatric population with CKD, depending on the clinical condition and the results of laboratory tests, as well as the type of treatment of RRT in pharmacotherapy, the following drugs (in individual doses), were used: antihypertensive drugs (e.g., calcium channel blockers, angiotensin-converting enzyme inhibitors—ACE inhibitors, β-blockers), vitamins: D_3_, C, B, folic acid, proton pump blockers, erythropoietin, calcium carbonate, iron preparations.

Control group (Group I)—Thirty-three healthy children (14 boys, 19 girls) with normal kidney function. These children were hospitalized in the Department of Pediatric Nephrology of the University Hospital due to suspected dysfunctions of the lower urinary tract (mainly nocturnal enuresis). On the basis of diagnostic tests carried out at that time, the above-mentioned abnormalities were excluded. None of these patients were diagnosed with a chronic disease nor were they treated with specialist pharmacotherapy.

The biochemical parameters in the CKD children and control group are presented in Table 1.

### 2.2. Ethical Issues

The research project has been approved by the Bioethics Committee of the Wrocław Medical University, issue KB-369/2017 of 6 June 2017.

All procedures involving human participants were in accordance with the highest ethical standards of the institutional research committee and were performed according to the Declaration of Helsinki on the treatment of human subjects and its later amendments. Both the children of all groups participating in the study (≥16 years) and their parents were informed about the aims, principles, benefits and informed consent was obtained.

## 3. Methods

### 3.1. Collection of Test Material Samples

Blood samples from selected pediatric patients were collected in the morning in the sitting position, from the ulnar vein, during the planned diagnostic and therapeutic procedures. In dialysis patients, blood was collected between hemodialysis sessions. The material was collected from fasting patients in two ways: whole blood collected into test tubes with EDTA-K and into test tubes with citrate as an anticoagulant. Samples were prepared according to the procedure described in Section 3.3 for the determination of nucleotide metabolites. The samples were stored in a freezer at −80 °C until the liquid chromatography-mass spectrometry (LC-MS/MS) analysis was performed.

### 3.2. Reagents

Water, acetonitrile and methanol (Merck); NAMN, NMN, NAAD, NA, BCl and PCA (perchloric acid) (Sigma, Saint Louis, MA, USA); NAM, NAD and NADH (Koch Light Laboratory GmbH) were used in the research. The research was carried out in the Chair and Department of Medical Biochemistry of the Medical University and in the Mass Spectrometry Laboratory of the Technology Park (WTP) [18].

### 3.3. Preparation of Samples for the Determination of Nucleotide-Related Metabolites

The analysis of samples taken for the determination of adenine nucleotide-related metabolites (NAD, NADH, NA, NAM, NMN, NAMN, NAAD) was carried out on red blood cells obtained from whole blood collected on EDTA. Procedure for patient samples: 400 μL of whole blood (1000× *g*, 5 min, 4 °C) was centrifuged. Plasma and buffy coat were discarded, blood cells were flushed twice with 0.9% NaCl and counted (applied dilution of blood cells; 1:1 trypan blue stain). As much as 50 μL of erythrocytes were added to 400 μL of 0.5 N perchloric acid (PCA), shaken (5 min, 800 g) and centrifuged (15,000× *g*, 10 min, 4 °C). Samples were kept on ice for 30 min at −20 °C. In the next step, 300 μL of supernatant was removed. Before measurements, 100 μL of supernatant was diluted in 0.1% formic acid (FA) solution in water. In the case of standards, 400 μL of 0.5 N PCA was added to 100 μL of the standard solution. Subsequently, the samples were shaken (5 min, 800 g), centrifuged (15,000× *g*, 10 min, 4 °C) and then placed on ice for 30 min at −20 °C. As much as 100 μL of 0.1% FA solution in water was added to 100 μL of the supernatant obtained.

### 3.4. Method of Analysis for Nucleotide Metabolites

In order to analyze metabolites in the sample at the same time, it was necessary to develop a method for their determination. The analysis time for an individual sample was 13 min and the volume of the sample for analysis was only 50 μL of erythrocytes. By using the appropriate concentration and elution time of the mobile phase during the chromatographic separation (Table 2), it was possible to separate individual nucleotide metabolites.

The chromatographic separation of adenine nucleotide-related metabolite samples was performed with Acquity UHPLC liquid chromatograph with a cooled autosampler and Acquity HSS T3 column (50 m × 1.0 mm, 1.75 µm), coupled with Xevo G2 Q-TOF mass spectrometer (Waters). The separation time of a single injection of 2 µL was 13 min and the mobile phase flow was 95 µL/min. For gradient elution (Table 2), 10 mM ammonium acetate with 0.1% FA in water was used as phase A and 10 mM ammonium acetate with 0.1% FA in methanol was used as phase B. In the spectrometer, the ion source was of electrospray ionization (ES) type, operating in positive ion mode. The samples were ionized using the ESI. The source parameters were optimized to obtain the highest sensitivity for the tested compounds. The electrode voltage was 4500 V. The other parameters of the ion source were set as follows: ion source temperature: 110 °C; desolvation temperature: 350 °C. Nitrogen was used as a nebulizing and drying gas. Quanlynx software (Waters) was applied to collect and process the data. The following ions were used for quantitative analysis: 123.0553 *m/z (mass-to-charge ratio)* for NAM; 124.0393 *m/z* for NA; 664.1169 *m/z* for NAD; 666.1169 *m/z* for NADH; 335.0644 *m/z* for NMN; 336.0484 *m/z* for NAMN; 665.1010 *m/z* for NAAD.

**Figure 1:**
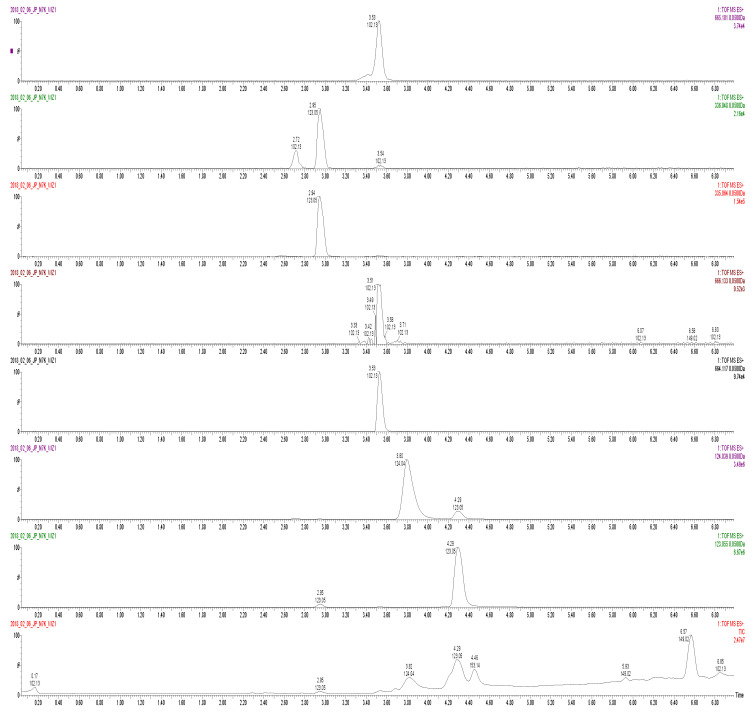
Total ionic current (TIC) chromatogram for patient exemplified sample. Data represent single ion current chromatograms for particular metabolites (NAAD, NAMN, NMN, NADH, NAD, NA and NAM, from the top) from with their retention times on horizontal axis and total ion current on the bottom chromatogram for patient sample.

The chromatograms of NAD metabolites of sample from representative patient are shown in Figure 1.

The peak area ratio of the tested compound in relation to the peak area of the corresponding standard was used to perform linear regression analysis. A linear regression coefficient (r^2^) was calculated for each standard. For nucleotide metabolites r^2^ > 0.956.

### 3.5. Statistical Analysis

The results of the analyses were presented as mean (x), standard deviations (SD), median (M), lower and upper quartiles (25–75 Q). The equality of means in independent groups was tested with ANOVA analysis of variance. A nonparametric Mann–Whitney U test and the Kruskal–Wallis test were performed. Spearman’s correlation coefficient was used to analyze the relationships between the tested parameters. Statistically significant differences at *p* < 0.05 level were assumed. A statistical analysis was performed using STATISTICA 8 software (StatSoft).

## 4. Results

### 4.1. Comparison of the Results of Determinations between the CKD Children and Control Group

The results of all statistical analyses for the determination of nucleotide metabolites in erythrocytes are presented in relevant tables. A detailed representation of the mean, standard deviation and median for each parameter is shown in Table 3.

The higher concentrations (although not statistically significant) of nucleotide were observed in the control group for NAD, NA, NAAD, NADH, NAMN, NMN in relation to CKD children. Only in the case of NAM, lower concentrations of this compound (without statistical significance) were found in the group of healthy children (mean 242.39 ± 204.04 nmol/mL), whereas the mean in CKD patients was 298.56 ± 238.78 nmol/mL.

### 4.2. Comparison of the Content of Nucleotide-Related Metabolites with the CKD Severity

Differences between individual stages of CKD are only for NAD. NAD concentrations reached the lowest values in Group II patients.

Statistically significant differences in NAD concentrations were observed between CKD patients with stages II–IV and III–IV.

On the other hand, in CKD patients, the highest NAD concentrations were observed in Group IV (mean 324.94 ± 63.06), which was statistically significantly different in relation to Group II (*p* = 0.032) and Group III (*p* = 0.045).

Table 4 shows average NAD concentration values of erythrocytes for individual stages of CKD and control (nmol/mL).

Similar correlations are also found for other nucleotides, i.e., higher concentrations (although not statistically significant) were observed in the control group for NA, NAAD, NADH, NAMN, NMN in relation to CKD children. Only in the case of NAM, were lower concentrations of this compound (without statistical significance) found in the group of healthy children (mean 242.39 ± 204.04 nmol/mL), whereas the mean in CKD patients was 298.56 ± 238.78 nmol/mL.

Table 5 shows the average values of NA, NAM, NAAD, NAMN and NMN concentration in erythrocytes for individual stages of CKD and control group (nmol/mL).

### 4.3. Assessment of Dependence in the Groups of Children with CKD

The test results showed the following correlations in the groups of children with CKD:

Positive correlations of statistical significance between:

- NAD and NAAD (r = 0.852, *p* = 0.001),
and NAMN (r = 0.564, *p* = 0.001),
and NMN (r = 0.641, *p* = 0.001),
and with NADH (r = 0.850; *p* = 0.001).
- NAAD and NAMN (r = 0.677, *p* = 0.001),
and NMN (r = 0.742, *p* = 0.001)
and with NADH (r = 0.765, *p* = 0.001).
- NAMN and NMN (r = 0.874, *p* = 0.001)
and with NADH (r = 0.542, *p* = 0.001)
- NMN correlated positively with NADH (r = 0.585, *p* = 0.001)

NAD did not show any correlation with NAM in any study group.

## 5. Discussion

Maintaining an adequate physiological concentration of ATP/nucleotides in RBC affects cell life expectancy [19]. These processes are undoubtedly intensified during metabolic diseases, chronic inflammation, and in the case of chronic kidney disease, they are co-responsible for eryptosis.

Our observations revealed that in CDK children there are no obvious red blood cell metabolism disorders regarding the metabolites of adenine nucleotides. In contrast, erythrocytic NAD concentration shows significant differences between stage II and IV as well as stage III and IV of the disease. This indicates the activation of the defence mechanisms, leading to metabolic/energy stabilization of the erythrocyte.

NAD plays a fundamental role in energy reactions, but also in other basic processes of cell signaling, gene expression or DNA repair [20,21,22]. According to many researchers, the level of adenosine triphosphate (ATP) in red blood cells depends on the proper course of glycolysis, and thus on the appropriate concentration of NAD [20,21,22,23].

Hikosaka et al. additionally showed that the blockade of the glycolytic pathway in red blood cells (RBC) occurred at the stage of glyceraldehyde-3-phosphate dehydrogenase (GAPDH) due to lack of NAD coenzyme [4]. These observations revealed the unexpected role of Nmnat3 in maintaining the appropriate NAD concentration in erythrocytes and related regulation of RBC lifespan [4].

A number of studies have been undertaken on the occurrence of abnormalities in the metabolism of compounds involved in glycolysis, and thus in the basic physiological processes. In 6-phosphate dehydrogenase (G6PD) deficiency, decreased NADPH regeneration in the pentose phosphate pathway and diminished levels of reduced glutathione cause insufficient antioxidant defenses, increased sensitivity of red blood cells to oxidative stress and acute hemolysis after exposure to pro-oxidants and inflammation [4,24].

In mammals, NAD is synthesized from various sources. Its main precursors include tryptophan (Trp), nicotinic acid (NA), nicotinamide riboside (NR), nicotinamide mononucleotide (NMN) and nicotinamide (NAM). Based on the bioavailability of these precursors, there are three pathways for the synthesis of NAD in cells: from Trp through the de novo biosynthesis pathway or the kynurenine pathway; from NA in the Preiss–Handler path; and with NAM, NR and NMN in the rescue path [25]. Maintaining NAD homeostasis as a response to environmental factors or stimuli highlights NAD activities in coordinating metabolic reprogramming and maintaining physiological cell biology. Hence, NAD and its metabolites serve as a metabolic center in both physiological and pathophysiological processes. They may also represent future therapeutic potential in NAD modulation in the treatment of metabolic diseases, neurodegenerative and oncological diseases.

In turn, NAAD and NAD initially show in stage III of the disease a downward trend in concentrations (without statistical significance) and already in stage III–IV an increase is observed in their cellular level. In the case of uremic red blood cells, NAD fluctuations are statistically significant in stages II to IV of the disease. Although the studied nucleotide NAM does not show statistically significant changes in uremic blood cell levels, it nevertheless has an interesting (and thought-provoking) “course” of concentrations in the progression of CKD. The trend of these changes is different in relation to the remaining metabolites of adenine nucleotides analyzed. In stage II of the disease, it shows the highest value among the other stages of CKD, reaching the lowest concentration value in stage III. Taking into account the rescue pathway of the NAD cellular synthesis, we can consider that the observed growth fluctuation in stage III of NAD disease results from, among others, the use of NAM as a substrate. In CKD patients, increased activation of enzymes affecting NAM metabolism, including poly(ADP-ribose) polymerase, is also possible. The cytoprotective properties of NAM are associated with the inhibition of poly(ADP-ribose) polymerase activity.

An interesting phenomenon was noted by us regarding the statistically insignificant fluctuation tendency of concentration of metabolites NA, NAM, NAAD, NAMN and NMN for stage III CKD. All mentioned parameters had either the lowest or one of the lower cellular levels in the uremic RBC. Patients with stage IV CKD had higher concentrations of NAD than patients in stage II and III, however, no difference was noted in comparison to control group and between other groups. Given relatively small sample sizes of subgroups, it is possible that type II error has occurred. Further studies are needed to establish the relationship between NAD levels CKD stages and RRT. In the course of chronic kidney disease progression, each stage of the disease has more or less expressed “newly” attached metabolic disorders. In the pediatric population, the body maintains homeostasis for a relatively long time, through a variety of repair processes. Such stabilization takes place especially in the first two stages of CKD. From a practical point of view, stage III is a clear beginning of the clinical manifestation of multifaceted metabolic disorders of the body, including the first symptoms of uremic anemia, which are the result of a shortened period of life of the red blood cell. Hikosaka’s team published a study linking NAD and NADH metabolism insufficiency in erythrocytes with splenomegaly and hemolytic anemia in mice [4].

These concentrations were significantly reduced compared to those of the control group. For other NAD-associated metabolites, these authors recorded slight decreases for NAM and NMN in erythrocytes in mice with hemolytic anemia. Hikosaka and coworkers believe that the reduced concentration of NAM and NMN may be caused by an impairment of the “recovery process” in erythrocytes [4]. On the basis of results, we are inclined to agree with this conclusion.

The pre-dialysis stage of the disease (i.e., stage IV CKD) at the cellular level is characterized by the highest concentrations for NAM, NAAD, and NAD and NADH. Only for NAD concentration are the values statistically significant compared to those of stage II and III of the disease. At the same time, for NA, NAMN, NMN, cellular concentrations remain at a level similar to those of the physiological erythrocytes of the control group. Stage IV CKD is the culmination of the biochemical repair processes taking place in the cell undergoing increasing uremic toxemia.

Red blood cell red-ox homeostasis requires a continuous supply of energy, i.e., maintaining a sufficiently high concentration of erythrocytic adenosine triphosphate (ATP_e_), and energy potential (energy charge) inside the cell. The level of cytoplasmic ATP_e_ and the “energy charge” values of erythrocytes are determined not only by the rate of synthesis in the process of anaerobic glycolysis and the process of reutilization of adenyl nucleotides. In our 2012 study also conducted on CKD children, we observed a gradual increase in ATP_e_ concentration from stage I-III CKD [26]. In the pre-dialysis stage, however, there was a large decrease in the concentration of erythrocytic ATP_e_. The reason for such a change in ATP concentration is probably a decrease in the activity of lactate dehydrogenase (LDH) as well as the “redirection” of glucose catabolism towards the pentose-phosphate pathway. Such a direction in erythrocytic metabolic disorders is confirmed by our subsequent research also presented in the aforementioned publication. In the process of the erythrocyte anaerobic glycolysis, a special role plays LDH, whose activity and direction of action is decisive for the rate of glycolysis and the associated energy consequences and red-ox of the cell. In the studied population of CKD children, we initially had a gradual increase in LDH activity to stage III of CKD. In the pre-dialysis period, however, there was a significant decrease in LDH activity in relation to the examined patients and in relation to the control group. The accompanying CKD hypoxia is also of importance; greater transport of lactates and ion H^+^ ions inside the cell as a result of, among others, inactivation of LDH [26].

Stressors such as ischemia induce enzymes consuming NAD such as poly-ADP-ribose polymerases (PARPs), and the induction of these enzymes lowers cellular NAD [27]. The above observations on the onset of metabolic disorders at the cell level are reflected in the clinical state of the CKD patient.

In the group of children treated with renal replacement for NA and NMN, the lowest cellular concentrations in the course of the disease were found, but without signs of statistical significance. The other metabolites NAM, NAAD, NAMN and NAD present concentrations similar to those found in a healthy population. Such a metabolic state of red blood cells allows us to suppose that renal replacement treatment brings the cells closer to biological physiology. However, the question remains of the duration of dialysis therapy and its secondary metabolic complications.

Although the results obtained in the current publication did not identify clearly statistically significant disturbances in the erythrocytic concentrations of adenine nucleotide metabolites besides NAD, the observations made seem to be helpful in better understanding the relationship between the metabolism of individual parameters involved in different biochemical cycles, and above all its energy level. In addition, they shed more light on the complex pathomechanism of metabolic profiles of chronic kidney disease. Maintaining physiological (low levels) oxidative stress, also referred to as oxidative eustress, is critical in regulating biological processes and physiological functions, including the RBC cell cycle [28,29]. From a clinical point of view, according to Na Xie’s team, it is important that the new NAD red-ox regulation through sirtin -3 (SIRT3) dependent deacetylation in response to oxidative stress, improve resistance to the harmful effects of oxidative damage [23].

On the basis of the performed experiments in this paper, we agree with the opinion of Benito and co-workers that a multivariate analysis of data on plasma concentration of metabolites of the urea cycle, arginine methylation and metabolic pathways of arginine and creatinine in pediatrics makes it possible to “classify” a child to a specific stage of the disease with 74% accuracy with currently up to 90% of the diagnoses performed by doctors (one stage above or below) regarding the advancement of CKD being erroneous [2,3]. Therefore, further metabolomic studies are necessary and will undoubtedly contribute to the identification of disorders and later therapeutic implications.

Considering the overall design of our observation, it should be emphasized that this is the first study of CKD children regarding adenine nucleotide metabolites. However, this study has limitations. We are aware that it would be necessary to analyze more patients. It would also be useful to expand the study group to include children who have had a kidney transplant. Investigation of the correlations of NAD metabolites concentrations with other erythrocyte characteristics such as size and hemoglobin content should be also performed.

## 6. Conclusions

1. CKD children do not have evident abnormalities of RBC metabolism with respect to adenine nucleotide metabolites.
2. The significant differences in erythrocyte NAD concentrations between CKD stages may suggest the activation of adaptive defense mechanisms aimed at erythrocyte metabolic stabilization.
3. It seems that the implementation of RRT has a positive impact on RBC NAD metabolism, but further research performed on a larger population is needed to confirm it.

## Figures and Tables

**Table 1:** The biochemical characteristics of the blood of studied population of patients.

Material	Studied Parameter	Study Group/Patient Group		Control Group		Significance Level *p*-Value
		N	Mean ± SD	N	Mean ± SD	
blood	GFR (mL/min/1.73 m^2^)	48	45.96 ± 26.81	33	103.15 ± 10.88	0.0001
	creatinine (mg/dL)	48	2.25 ± 1.99	33	0.56 ± 0.11	0.0001
	inorganic phosphorus (mg/dL)	44	4.94 ± 0.84	33	5.36 ± 1.17	0.194 (NS)
	calcium (mg/dL)	43	9.80 ± 0.68	32	10.04 ± 0.47	0.020
	sodium (mg/dL)	48	139.46 ± 3.35	33	138.94 ± 2.73	0.381 (NS)
	potassium (mg/dL)	48	4.47 ± 0.46	32	4.42 ± 0.39	0.643 (NS)
	urea (mg/dL)	47	60.85 ± 38,91	32	26.16 ± 15.86	0.0001
	Hb (g/dL)	48	11.7 ± 1.51	34	14.4 ± 1.74	0.0001
	Ht (%)	48	34.33 ± 4.15	34	41.38 ± 4.88	0.0001
	RBC (*10^6^*/*µL)	48	4.14 ± 0.71	34	4.85 ± 0.55	0.0001

Legend: NS—not significant; SD—standard deviation.

**Table 2:** Percentage gradient distribution of mobile phases during chromatographic separation of LC-MS/MS for nucleotide-related metabolites (phase A—10 mM of ammonium acetate with 0.1% FA in water, phase B—10 mM ammonium acetate with 0.1% FA in methanol).

Time [min]	Phase A [%]	Phase B [%]
0	99	1
2.7	0	100
5.7	0	100
5.71	99	1
13	99	1

**Table 3:** Comparison of test results for both study and control group (N = 81).

Material	Studied Parameter	Study Group/Patient Group			Control Group			Significance Level *p*-Value
		N	Mean ± SD	Median (Q25 Q75)	N	Mean ± SD	Median (Q25 Q75)	
erythrocytes	NAD	48	216.98 ± 117.87 ^N^	222.48 (119.84–317.8)	33	233.30 ± 113.11	256.08 (177,60–289.12)	0.269 (NS)
	NA	47	8.69 ± 5.08 ^N^	7.84 (5.44–10.24)	33	9.04 ± 4.66	8.00 (6.40–11.52)	0.756 (NS)
	NAM	46	298.56 ± 238.78	171.04 (132.16–510.72)	33	242.39 ± 204.04	150.64 (139.6–183.52)	0.183 (NS)
	NAAD	47	119.29 ± 73.45 ^N^	121.92 (49.04–188.00)	33	136.40 ± 69.60	156.00 (86.40–172.24)	0.350 (NS)
	NAMN	47	40.00 ± 8.61	38.56 (33.28–44.40)	33	41.28 ± 10.30	38.00 (33.76–46.48)	0.809 (NS)
	NMN	47	40.90 ± 9.75	40.08 (33.52–48.16)	33	43.53 ± 10.68	41.44 (34.80–49.84)	0.350 (NS)
	NADH	47	92.38 ± 53.66 ^N^	106.64 (32.64–129.44)	33	105.61 ± 59.30	101.84 (62.72–146.72)	0.273 (NS)

Legends: NS—non-significant; the index ^N^ means that the studied parameter has the normal distribution; SD—standard deviation.

**Table 4:** Differences between stages of CKD severity for NAD.

Variable	NAD		Multiple Comparisons *p* Values (with a Bonferroni Adjustment)				
Stages of CKD	N	Median (Q25 Q75)	Control	II	III	IV	RRT
Control	33	256.08 (177.6–289.12)		1.000	1.000	0.148	1.000
II	15	201.6 (78.64–303.28)	1.000		1.000	0.032	1.000
III	16	221.12 (144.84–261.84)	1.000	1.000		0.046	1.000
IV	8	340.52 (315.88–353.28)	0.148	0.032	0.046		0.194
RRT	9	252.00 (23.44–304.88)	1.000	1.000	1.000	0.194	

Legends: RRT—renal replacement therapy; C (I)—control; II, III, IV—stage of CKD.

**Table 5:** Median of NA, NAM, NAAD, NAMN, NMN and NADH concentration values for individual stages of CKD and control group (nmol/mL).

Variables	NA		NAM		NAAD		NAMN		NMN		NADH	
Stages of CKD	N	Median (Q25 Q75)	N	Median (Q25 Q75)	N	Median (Q25 Q75)	N	Median (Q25 Q75)	N	Median (Q25 Q75)	N	Median (Q25 Q75)
Control	33	8.00 (6.40–11.52)	33	150,64 (139.6–183.52)	33	156.00 (86.40–172.24)	33	38.00 (33.76–46.48)	33	41.44 (34.8–49.84)	33	101.84 (62.72–146.72)
II	14	8.08 (5.44–9.76)	14	202.52 (147.52–566.88)	14	117.60 (44.08–178.16)	14	38.88 (34.56–45.92)	14	40.44 (34.32–49.36)	14	97.36 (27.20–121.52)
III	16	7.72 (5.40–10.44)	15	136.56 (128.88–510.72)	16	120.64 (61.72–152.76)	16	37.28 (33.24–42.84)	16	40.24 (33.56–47.52)	16	74.56 (39.16–110.92)
IV	8	8.76 (6.44–15.60)	8	177.48 (151.64–362.32)	8	192.88 (66.40–212.00)	8	38.64 (31.64–45.16)	8	40.52 (32.00–50.80)	8	125.88 (116.56–181.04)
RRT	9	6.32 (3.36–8.72)	9	159.52 (138.64–184.4)	9	141.76 (16.24–188.00)	9	42.24 (33.92–47.84)	9	38.56 (31.28–48.08)	9	97.92 (26.00–126.56)

Legends: RRT—renal replacement therapy; C (I)—control; II, III, IV—stage of CKD. (The Kruskal–Wallis H test with a Bonferroni adjustment showed no statistically significant differences between the various stages of CKD).
